# A Novel Surgical Treatment Management Algorithm for Elbow Posterolateral Rotatory Instability (PLRI) Based on the Common Extensor Origin Integrity

**DOI:** 10.3390/jcm13082411

**Published:** 2024-04-20

**Authors:** Christos Koukos, Michail Kotsapas, Konstantinos Sidiropoulos, Aurélien Traverso, Kerem Bilsel, Fredy Montoya, Paolo Arrigoni

**Affiliations:** 1 Medical Center Wuppertal, 42329 Wuppertal, Germany; koukos_christos@hotmail.com; 2 Sports Trauma and Pain Ιnstitute, 54655 Thessaloniki, Greece; 3 Orthopaedic Department, General Hospital of Naousa, 59200 Naousa, Greece; 4 Medical School of Patras, University of Patras, 26504 Patras, Greece; 5 Emergency Department, Papageorgiou General Hospital of Thessaloniki, 54635 Thessaloniki, Greece; 6 Orthopaedic Department, Centre Hospitalier Universitaire Vaudois (CHUV), 1011 Lausanne, Switzerland; 7 ASST Pini-CTO, 20122 Milan, Italy; 8 Faculty of Medicine, Acibadem Mehmet Ali Aydınlar University, 34752 Instanbul, Turkey; kerem.bilsel@acibadem.com; 9 Orthopaedics and Traumatology Department, FulyaAcibadem Hospital, 34349 Instanbul, Turkey; 10 Sanatorio Aleman Clinic, Universidad de Concepcion, Concepcion 4070386, Chile; fmontoya@puc.cl

**Keywords:** posterolateral rotational instability (PLRI) of elbow, PLRI diagnosis, clinical tests for elbow instability, common extensors origin (CEO) integrity, PLRI surgical treatment algorithm

## Abstract

**Background**: Here, we introduce a comprehensive treatment algorithm for posterolateral rotatory instability (PLRI) of the elbow, a condition affecting elbow mobility. We outline a diagnostic approach and a novel surgical management plan through the arthroscopic surgeon’s point of view. **Methods**: The central focus of this management approach is the integrity of common extensor origin (CEO). High clinical suspicion must be evident to diagnose PLRI. Special clinical and imaging tests can confirm PLRI but sometimes the final confirmation is established during the arthroscopic treatment. The most appropriate treatment is determined by the degree of CEO integrity. **Results**: The treatment strategy varies with the CEO’s condition: intact or minor tears require arthroscopic lateral collateral ligament imbrication, while extensive tears may need plication reinforced with imbrication or, in cases of retraction, a triceps tendon autograft reconstruction of the lateral ulnar collateral ligament alongside CEO repair. These approaches aim to manage residual instability and are complemented using a tailored rehabilitation protocol to optimize functional outcomes. **Conclusion**: PLRI is a unique clinical condition and should be treated likewise. This algorithm offers valuable insights for diagnosing and treating PLRI, enhancing therapeutic decision-making.

## 1. Introduction

The elbow is a congruent joint presenting inherent stability due to bone morphology (ulnohumeral joint) and anatomic restraints, both static and dynamic. The ulnohumeral joint serves as a primary static stabilizer, with the medial collateral ligament (MCL) and the lateral collateral ligament complex (LCLC) as the major stabilizing ligaments [1,2]. Instability arises from the insufficiency of these structures due to trauma or degeneration. A significant lesion that leads to the posterolateral rotatory instability (PLRI) of the elbow is a dysfunction of the ulnar component of the lateral collateral ligamentous complex (LUCL) [3,4,5,6]. PLRI was first described by O’Driscoll et al. [7,8], and is the most common form of chronic elbow instability. This entity is characterized by a wide range of clinical manifestations, ranging from minor laxity to gross instability and elbow dislocations [3,9]. Consequently, addressing PLRI is challenging and entails a thorough physical examination, adequate imaging, and meticulous preoperative planning. Even though the importance of the common extensor origin (CEO) as a secondary lateral static stabilizer has been highlighted in the literature [10], to our knowledge, there is a lack of clarity on whether its condition should be taken into consideration systematically. This review strives to elucidate the pathophysiology leading to PLRI and to suggest a PLRI management algorithm that takes CEO integrity into account.

## 2. Anatomy and Considerations

The condition known as posterolateral rotatory instability (PLRI) was described by O’Driscoll et al. in 1991 as an instability pattern of the elbow that results from an weakened lateral ulnar collateral ligament (LUCL), which is a restraint to varus stress and acts to stabilize the radial head from posterior subluxation or dislocation [8]. Recent studies suggest the potential for broader compromise within the lateral collateral ligamentous complex [11,12]. Its etiology is mainly a consequence of trauma in 94% of patients, involving either an elbow dislocation or a fall with outstretched and supinated forearm [7,13,14]. A common mechanism is a fall onto an outstretched hand, leading to axial loading and elbow supination [4,15,16,17,18,19]. Minor repetitive microtrauma during sports or everyday activities also applies a substantial amount of stress on the LUCL, and if not addressed adequately results in the ligament’s attenuation and dysfunction, and ultimately PLRI [2]. Iatrogenic lesions, often a surgical complication, can also occur after multiple steroid injections during treatment for persistent lateral epicondylitis [2,15,20]. Excessive stripping of the lateral condyle during the open approach, aggressive arthroscopic or open approaches for the common extensor origin (CEO), or capsular release pose a risk of compromising the LCLC’s function due to its close anatomic proximity [2,10,15]. Repeated steroid injections affect the quality of both the CEO and the lateral stabilizers, leading consequently to CEO weakening and deficiency [10,19]. To summarize, it can be either an iatrogenic complication of continuous corticosteroid injections or of lateral epicondylitis surgery, as well as from cubitus varus injury due to the attenuation of ligaments.

Schnetzke M et al. [21] support that all simple elbow dislocations result in LCLC injuries, while magnetic resonance imaging (MRI) signal abnormalities in the CEO are evident in 39% of patients. Kim YS et al. [22] found that all patients with lateral epicondylitis present various degrees of CEO lesions and an LCLC dysfunction is present in 55%. They concluded that as the grade of the CEO lesion increases, so too does the incidence of potential PLRI in patients with lateral epicondylitis. The size of the CEO tear, the grade of CEO abnormality, and the presence of extensor muscle edema are the key factors related to the surgical management of lateral epicondylitis [23]. These data suggest a mutual relationship between CEO condition and LCLC integrity, both anatomically and functionally.

According to O’Driscoll, when the LUCL is not functional, the radial head rolls off the capitellum during forearm supination and subluxates posterolaterally. The ulnohumeral joint begins to gap and, as the pattern of instability continues, the entire elbow joint may dislocate. When the radial collateral ligament (RCL) does not heal properly and is nonfunctional, the radial head rolls off of the capitellum during forearm supination. As the radial head subluxates posterolaterally, the ulnohumeral joint begins to gap and, as the pattern of instability continues, the entire elbow joint may dislocate [8]. This concept has been debated recently throughout the literature demonstrating not a single dysfunctional lateral elbow anatomical structure but many of them, including radial collateral ligament, annular ligament (partially), and/or the common extensor mechanism [24]. Dunning et al. stated that to achieve PLRI, both the LUCL and the RCL must be sectioned. It has also been demonstrated that sectioning the anterior band of the lateral collateral complex induces instability, suggesting that an intact LUCL alone cannot stabilize the elbow [11,25]. Therefore, PLRI is not merely the result of an elbow dislocation but rather a range of injuries.

## 3. Clinical Presentation and Diagnosis

PLRI may have various clinical presentations with insidious courses of symptoms, making its diagnosis challenging to the inexperienced physician. Patients often complain of discomfort or lateral-sided pain during activities that require extension and supination, often associated with locking or catching during extension of the elbow at 40° flexion [8,24]. Most patients with chronic PLRI may have a normal appearance of the elbow with a full range of motion, and palpation may not trigger any pain [26].

Patients with chronic or subtle instability can be easily examined and assessed through clinical examinations such as the pivot shift test, the posterior radiocapitellar test, and the push-up and chair-rise tests [27]. It is crucial to note that the sensitivity of these tests heavily relies on the clinician’s experience and the patient’s tolerance [28]. Microinstability can be easily overlooked [9].

Plain X-rays are a necessity in the setting of acute elbow trauma to evaluate articular congruency and osseous injuries, while more complex cases may require investigation with Computed Tomography (CT) to reveal occult fractures or support preoperative planning [28]. Ultrasound (US) requires user experience and allows for real-time dynamic elbow examination [29,30]. Moreover, it is a very useful and inexpensive diagnostic tool to evaluate soft tissue integrity, particularly in lateral epicondylitis, with a reported sensitivity of 80% and a specificity of 50% [31]. According to a cadaveric study, US is superior to MRI in recognizing LUCL, demonstrating higher sensitivity and accuracy [6]. Nonetheless, MRI remains the modality of choice in soft tissue investigation [28]. Apart from ligament tears, scar tissue, or CEO ruptures, additional joint lesions, such as chondral lesions or osseous oedema, can also be evaluated with MRI [16,28]. Tendinous and ligamentous lesions are best evaluated in T2-weighted images [22,31,32]. In MRI, a radiocapitellar incongruity of more than 3.4 mm has an absolute positive predictive value for a pathology affecting elbow stability, while an incongruity of less than 0.3 mm has an absolute negative predictive value. In addition, a ulnohumeral incongruity of more than 1.5 mm has an absolute positive predictive value and less than 0.3 mm has a negative predictive value, excluding elbow instability, according to Hackl et al. [28]. A coexisting CEO injury correlates with increased incongruency on MRI [28]. Fluoroscopy under local anesthesia may show the radial head and proximal ulna posterolaterally subluxated and rotated [33].

The proximity of the LCLC and the CEO highlights the necessity of carefully evaluating the integrity of each structure if the other one is compromised. Bredella et al. advocate that lateral epicondylitis often co-occurs with LUCL lesions [32]. Furthermore, an acute traumatic LUCL rupture may be accompanied by a CEO rupture or avulsion [31,34]. According to Walz et al., CEO lesions in lateral epicondylitis are categorized as mild, moderate, and severe [31]. Mild lateral epicondylitis corresponds to CEO tendinosis or minor partial tears affecting less than 20% of the tendon thickness. Moderate lateral epicondylitis involves intermediate tears affecting more than 20% but less than 80% of the tendon thickness. Severe lateral epicondylitis is indicated by a high-grade partial-thickness tear (more than 80% of tendon thickness) or a full-thickness CEO tear [23,31,32].

## 4. Unique Features

Baker’s arthroscopic classification of CEO lesions was groundbreaking when it was published, but it has limited predictive value for patient outcomes [35]. We find an MRI-based, preoperative algorithm for classifying CEO integrity more beneficial as it determines the therapeutic course of action before the surgery, as opposed to during it. Treatment algorithms for acute and chronic elbow instability have been offered by Savoie [36] and van Riet [37], but they do not consider CEO integrity. O’Driscoll et al. [10] argue that if the CEO deficiency exceeds an undetermined limit, LCLC reconstruction may be subjected to high strain and a higher failure rate. We propose a treatment algorithm that addresses this issue, considering CEO quality without quantifying a threshold. The ideal approach for an arthroscopic surgeon is to formulate a preoperative plan using objective data from an MRI and confirm the diagnosis via arthroscopic assessment. These features are lacking in currently available protocols. A practical and straightforward management protocol is needed. Due to these limitations, we have developed and implemented a new treatment algorithm that emphasizes clinical features and only employs an open technique when absolutely necessary.

In summary, we aim to propose a PLRI treatment protocol that achieves the following:

1. It is straightforward to grasp.
2. It directly applies to clinical settings without requiring complex procedures.
3. It utilizes arthroscopic minimally invasive techniques, which minimize potential iatrogenic instability; in contrast, open procedures can exacerbate elbow instability, which is challenging to rectify [38].
4. It is contingent on the integrity of the CEO, whether it is intact, has a moderate or extensive partial tear, or is completely ruptured with or without retraction. This evaluation can be conveniently conducted via MRI.

PLRI is categorized into three stages. Stage 1 is characterized by the posterolateral rotatory subluxation of the elbow, which a pivot-shift test can confirm. Stage 2 involves an incomplete dislocation of the elbow, with the coronoid process positioned under the trochlea. In Stage 3, a full elbow dislocation occurs, moving the coronoid process behind the humerus. There are also three subcategories for Stage 3: 3A, where the MCL anterior band remains intact, allowing the elbow to remain stable against valgus stress post reduction; 3B, where the anterior band of the MCL is disrupted, leading to elbow instability under valgus stress post reduction; and 3C, where complete elbow instability is caused by the stripping of all the humerus’ soft tissues [8].

## 5. PLRI Treatment

MRI evaluation of CEO integrity is significant for the development of this proposed algorithm. Effective preoperative planning contributes to improved postoperative results. This treatment pathway can be determined after stress examination during arthroscopy [36], and postintervention arthroscopic instability evaluation can be repeated and compared to the preintervention examination.

**Figure 1:**
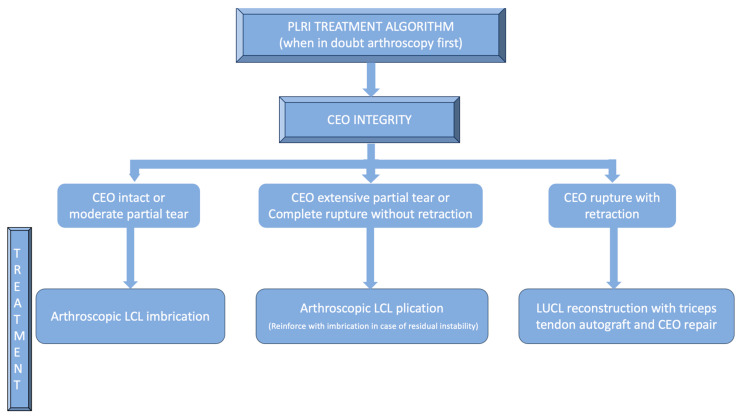
PLRI treatment algorithm according to CEO integrity.

**Figure 2:**
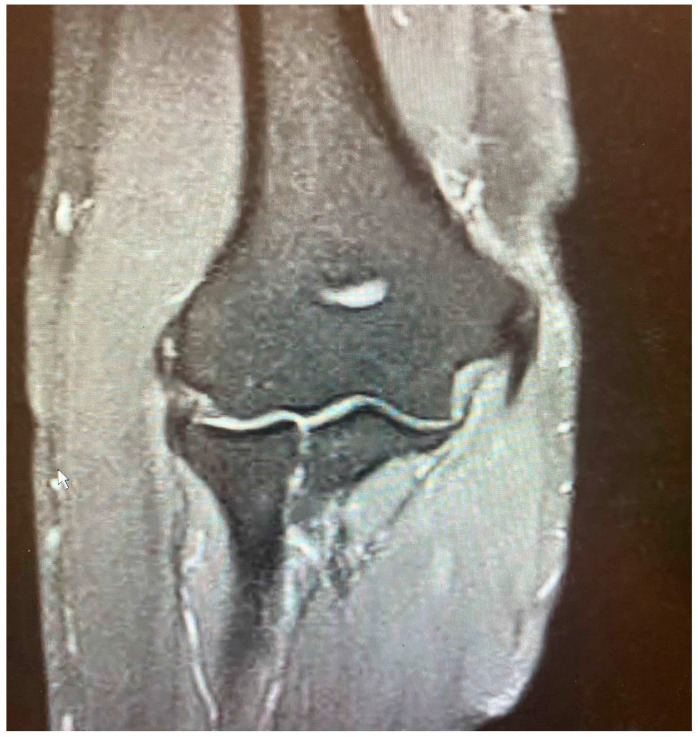
Minor partial CEO tear [courtesy of C.K.].

**Figure 3:**
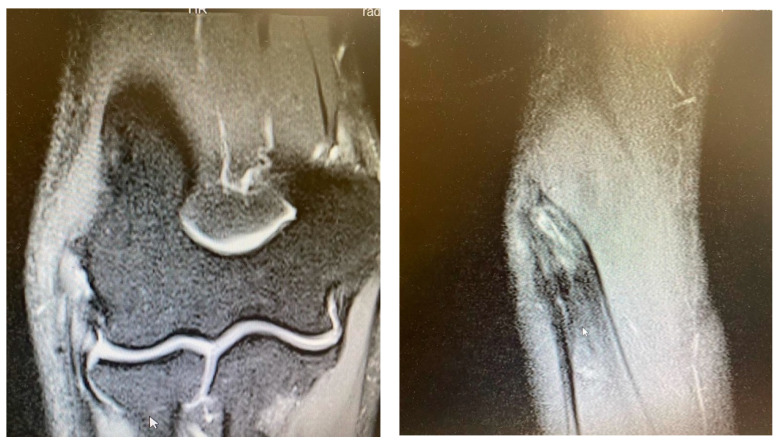
Extensive partial CEO tear [courtesy of C.K.].

**Figure 4:**
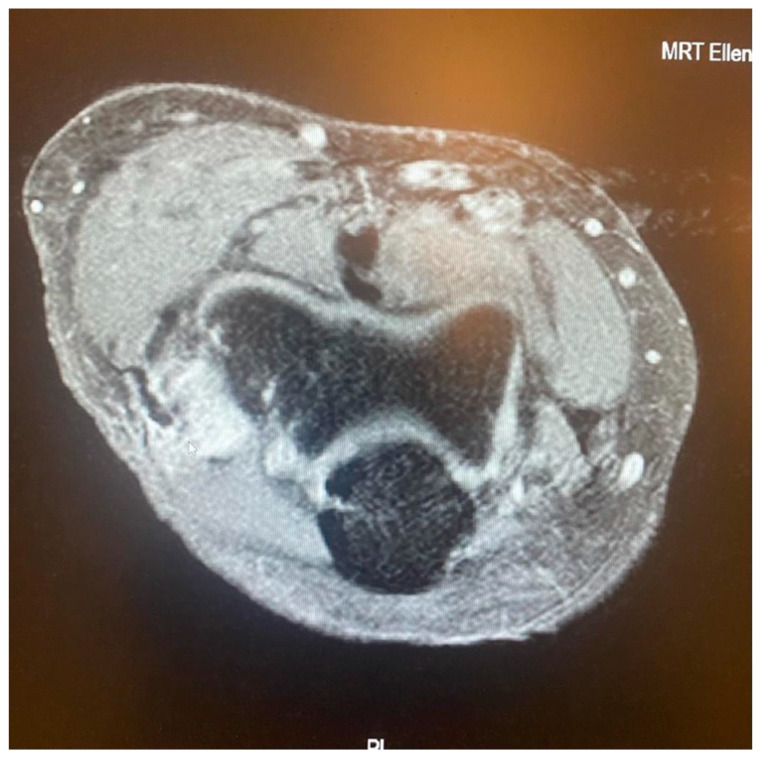
Complete CEO rupture [courtesy of C.K.].

**Figure 5:**
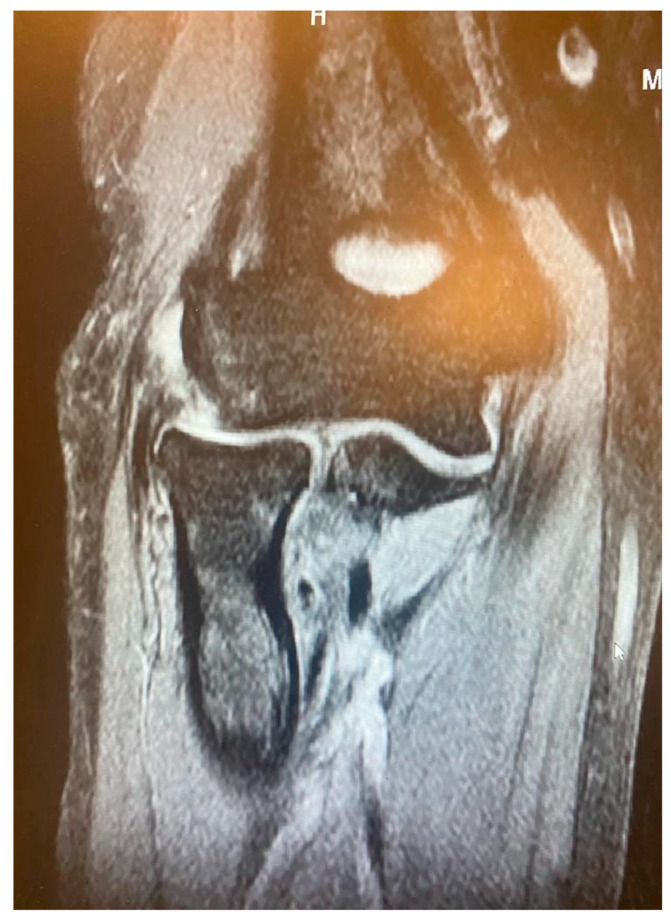
Complete CEO rupture without retraction [courtesy of C.K.].

**Figure 6:**
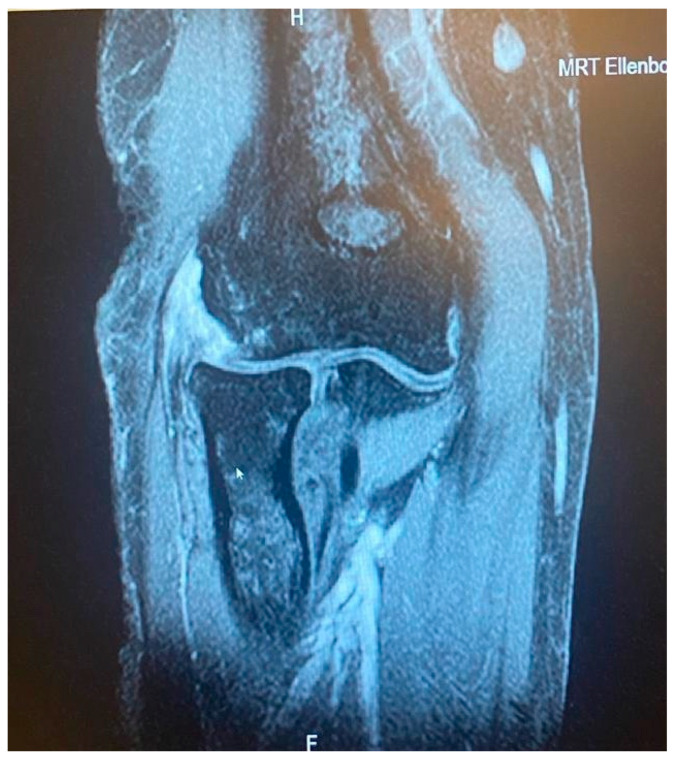
Complete CEO rupture with retraction and poor tendon quality [courtesy of C.K.].

While the Walz classification of CEO condition is useful, it has proven challenging to integrate directly into a treatment protocol, as it is designed specifically for diagnosing lateral epicondylitis. As such, we suggest a new integrity classification for the CEO that can better guide our PLRI treatment plan (Figure 1). First, PLRI must be confirmed; if not, we conduct an elbow arthroscopy for diagnosis. When the CEO is either intact or has a moderate partial tear, we propose arthroscopic LCL imbrication as the preferred treatment (Figure 2, Figure 3, Figure 4, Figure 5 and Figure 6). If there is extensive partial CEO rupture or complete rupture without retraction, arthroscopic LCL plication may yield good or excellent results. In cases where the CEO is ruptured and retracted, we recommend reconstructing the LUCL with a triceps tendon autograft and repairing the CEO through an open procedure.

Arrigoni et al. [9] evaluated pathologic lateral ligamentous laxity with the presence of three arthroscopic signs: the annular-drive through sign, the loose collar sign, and the pull-up sign of R-LCL (radial component of the lateral collateral ligament). In addition, any coexisting intra-articular pathology can be evaluated and addressed. Patients suffering from subtle instability may have an intact CEO or a moderate partial CEO tear on MRI. These patients may be managed with a minimally invasive soft tissue procedure, such as LCL imbrication [17]. This procedure offers a sufficient alternative option compared to open reconstruction surgery, with equivalent postoperative stability rates [3,17], and aims to regain tension of the lateral stabilizers, such as the LCLC, the lateral capsule, and the anconeus muscle [3]. The patient is first placed in the lateral decubitus position under sedation and regional anesthesia. Range of motion is documented with mobility maneuvers. Stability is evaluated using varus and valgus stress tests, pivot-shift, and posterior drawer maneuvers. A hemostatic cuff is placed on the proximal third of the arm and insufflated. A mark is placed at the center of the lateral epicondyle as well as one indicating the direction of the LUCL. The elbow is insufflated with saline solution.

The anterior compartment of the joint is visualized through an anteromedial portal. This allows us to demonstrate the opening of the radiohumeral joint by placing a feeler between the capitellum and the radial head. A high posterolateral portal is placed at the lateral tip of the olecranon to be able to visualize the posterior compartment, including the tip of the olecranon and the fossa (if any pathology is evident, a central posterior portal can be made to resolve said pathology). Valgus stress is applied to assess the integrity of the MCL under direct vision. In case the joint does not open, the MCL would be unharmed. Subsequently, the humerus–radial joint is visualized. The scope is pushed into the lateral gutter while a soft-spot portal is created to remove any synovium blocking the view. The arthroscopic rotatory instability test, the drive-through sign, and the trocar test, performed from the posterior compartment, help validate the diagnosis of PLRI [17]. Hypersupination with varus stress on the forearm will show the radial head subluxating posteriorly on the capitellum and the radiocapitellar joint line widening, while longitudinal pressure on the forearm may reveal more radiohumeral joint space gapping, the so-called pull test. An indirect indicator of lateral laxity is also the occasional sagging of the annular ligament [17].

A PDS II suture is threaded from the center of the epicondyle towards the radiohumeral joint using a suture passer (CHIA perc-passer, DePuy Synthes, Warsaw, IN, USA) and pulled out through the direct lateral portal with forceps. Another identical suture is directed towards the insertion of the LUCL in the radiohumeral joint from the subcutaneous edge of the ulna and retrieved via the same portal. This leaves two strands of PDS II sutures. The first enters the skin at the lateral epicondyle’s center, moves through the capsule and LCL complex, and exits through the direct lateral portal. The second does the same, starting at the ulna’s subcutaneous edge just below the LUCL insertion and passing through the anconeus, capsule, and LUCL. Both suture ends that exit through the direct lateral portal are tied together, forming a single suture line stretching from the lateral epicondyle to the ulna’s subcutaneous edge. Finally, the four suture ends are drawn out through the direct lateral portal.

The test for rotational stability is conducted once more, in which the forearm is fully supinated, and a forced varus is performed with both loose and tight sutures. After tightening the sutures, the rotatory instability test results turn negative, indicating that the sutures can be tied together. After the scope is removed, each suture is tied separately, and the knots are concealed. The portals are then closed, and a posterior splint is placed on the elbow. Patients are then fitted with a hinged brace. Postoperative radiographs or radioscopy are performed to ensure that the joint remains in reduction. After the initial postoperative visit, the elbow may be placed in a hinged elbow brace to allow for gradual movement (within a range of 30–120° for 4 weeks). Exercises for the peri-scapular region, wrist, and hand are permitted. Immediate physical therapy is mandatory.

**Figure 7:**
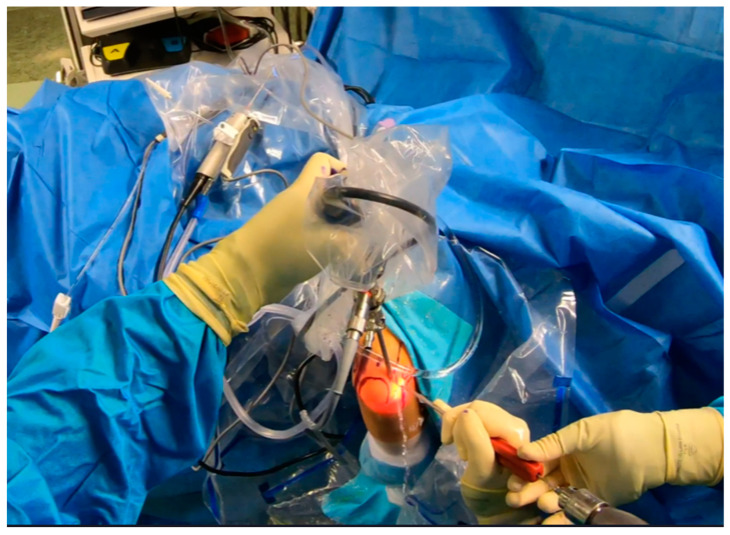
Arthroscopy. Drilling prior to anchor placement [courtesy of C.K.].

**Figure 8:**
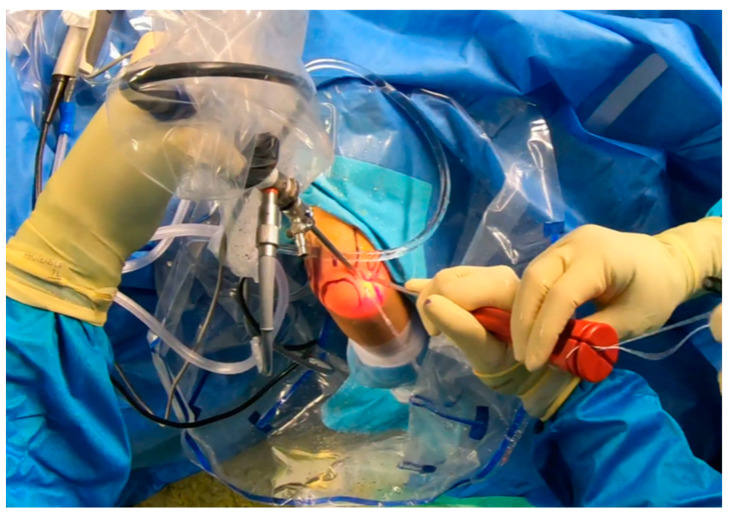
Anchor placement [courtesy of C.K.].

**Figure 9:**
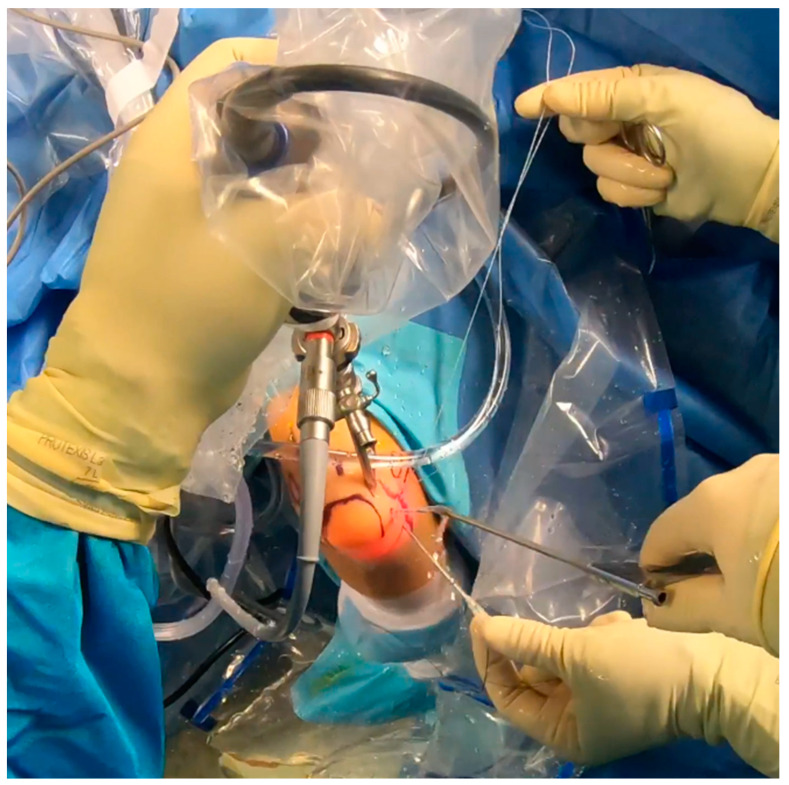
Suture shuttling by using a 14G needle [courtesy of C.K.].

**Figure 10:**
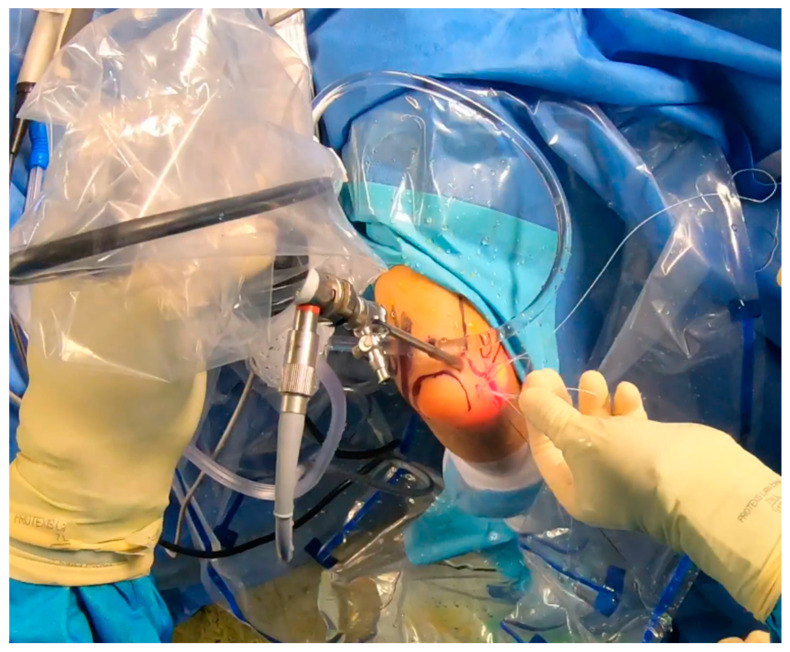
Suture shuttling (2nd stage) [courtesy of C.K.].

**Figure 11:**
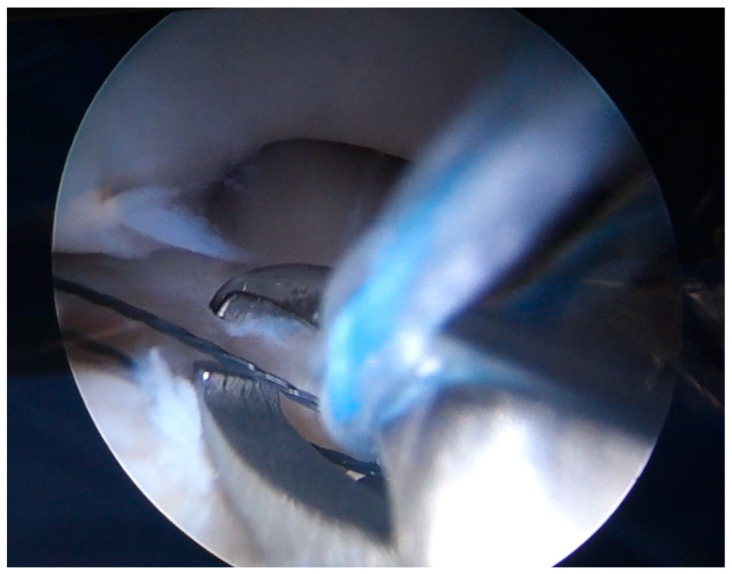
Arthroscopic view of suture shuttling [courtesy of C.K.].

**Figure 12:**
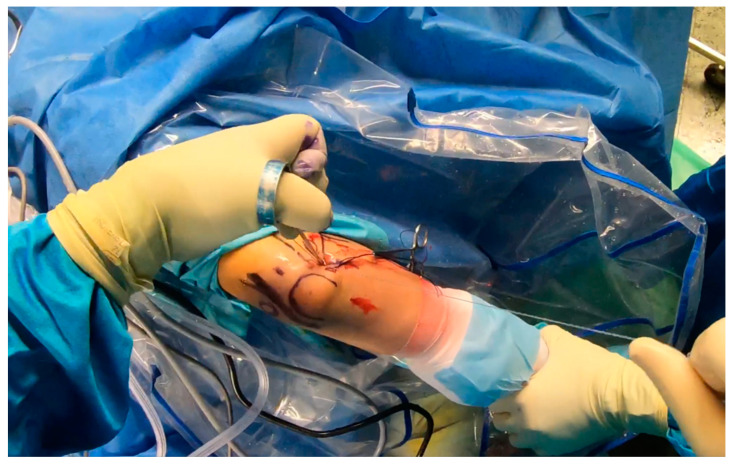
Final plication—suture tying by using sliding knots [courtesy of C.K.].

Patients with extensive partial or complete CEO ruptures, as evidenced by MRI scans, often see more benefits from an arthroscopic LCLC plication. This procedure, outlined by Savoie et al. [33], involves placing a double-loaded anchor at the isometric point on the capitellum’s lateral side. Then, sutures are placed into the posterolateral gutter from the radial border of the ulna, moving distally to the proximal. The first of these sutures is delivered through the annular ligament into the joint. Using a portal, the sutures are collected subcutaneously and tensioned and knotted as the arthroscope is removed from the lateral gutter. If any residual instability is noticed during arthroscopy post intervention, the plicated lateral soft tissues can be augmented with imbrication. After the procedure, the patient is fitted with a hinged brace for 4 weeks, allowing for neutral forearm rotation and an elbow extension range of 30–120°. Strengthening exercises are generally started after the sixth week post operation and continue until the third month. To allow for adequate healing, full weight-bearing activities should be avoided for 3 months post operation (Figure 7, Figure 8, Figure 9, Figure 10, Figure 11 and Figure 12).

Patients with a fully ruptured CEO and distal CEO retraction typically experience significant symptomatic instability. They need LUCL reconstruction with an autograft plus lateral epicondyle extensor repair [39]. Confirming PLRI via diagnostic arthroscopy should precede these procedures. Surgeons should carry out these interventions using an open procedure. As Dehlinger et al. outlined [40], the second step involves harvesting an ipsilateral triceps tendon autograft. This graft should measure at least 7 cm by 4.5 mm. The surgical technique from the primary author (C.K.) includes orienting the prepared graft with its ulnar side through a single cortical hole. This location should be at the annular ligament’s intersection with the radial head and neck, facing the distal ulnar direction to match the LUCL’s force vector. The graft’s humeral end is inserted at the humeral isometry point through a 2 cm deep hole made in the proximal–dorsal direction. The graft’s final ulnar position is secured with an anchor suture that also functions like a screw. The remaining suture then reinforces the fixation. On the humeral side, the graft’s position is secured with FiberWire^®^ (Arthrex, Naples, FL, USA), leaving a 2 cm intraosseous portion. Afterward, a tug on the graft secures it further with another suture anchor similar to a screw. Optimizing the humeral isometry point is next, following functional testing. The knotted FiberWire^®^ threads should be threaded into the screw for added support. The CEO is then reattached to the lateral epicondyle using anchor sutures. For large defects, surgeons can insert a suture anchor into the lateral condyle’s flexion side. After surgery, the patient wears a hinged brace set to allow extension/flexion from 30° to 120°. This continues for 4 weeks, followed by an additional 2 weeks without restrictions. The postoperative rehabilitation protocol includes extensor isometric exercises from the fourth week, followed by gradual weight training.

## 6. Discussion

Lateral elbow pain may be attributed to bony structure lesions (humerus, radius, and ulna), ligament lesions (LCLC), tendons (CEO), and nerves (branches of the radial nerve), making diagnostic and therapeutic management demanding [41,42]. PLRI and lateral epicondylitis may coexist, especially if lateral epicondylitis is persistent, misguiding the examiner [19,36]. As described by Arrigoni et al. [9], Symptomatic Minor Instability of the Lateral Elbow (SMILE) is a recently introduced clinical condition characterized by lateral ligamentous laxity usually associated with at least one intra-articular lesion, which can also mimic or accompany persistent lateral epicondylitis. Repeated corticosteroid injections or lateral elbow surgery for addressing lateral epicondylitis may raise clinical suspicion of lateral stabilizers’ compromise and potential PLRI in cases of persistent pain [20,34]. A thorough clinical and imaging investigation is required to avoid misdiagnosis. Since O’Driscoll first described PLRI as the result of LUCL insufficiency in 1991 [8], PLRI diagnosis has become more common [36] and various techniques for LCLC repair or reconstruction have been reported, as well as several modifications [2,17,36,37,41,43,44]. To the present day, there is no consensus on which treatment modality is more suitable for PLRI patients; nevertheless, some surgeons prefer arthroscopic techniques [3,17,41] and others open reconstruction [20,42].

For symptomatic patients with PLRI, surgical stabilization is recommended [20]. Regardless of the treatment method, the intention of re-stabilizing a joint is consistent; it aims to restore the form and functionality of the originally injured ligament. This study presents anatomical considerations for lateral-elbow-stabilizing structures, emphasizes the significance of the CEO in PLRI, and proposes a new treatment algorithm rooted in CEO integrity. The thought behind opting for arthroscopic LCL imbrication in patients with PLRI and either intact or moderately torn CEO is that open ligament reconstruction can be seen as an aggressive intervention, even for high-demand patients like professional athletes [29]. While open LUCL reconstructions provide impressive stability outcomes, the issues of extensive lateral soft tissue manipulations, scarring, and demanding postoperative rehabilitation protocols are noteworthy [3]. A well-known complication of LCL imbrication is subcutaneous irritation caused by suture knots [3], and suture sinking is recommended to decrease the irritation rate.

For patients suffering from partial CEO ruptures that are more extensive or complete ruptures without tendon retraction, isolated LCL imbrication may not prove sufficient. Therefore, if the remaining soft tissue quality allows it, a more suitable option would be the plication reconstruction of LCLC [33]. The critical component is incorporating the CEO into the plicated soft tissue so it can be used to repair the ruptured extensors concurrently. In the augmentation process involving LCL imbrication, the anconeus is included in the construct, which enhances the tension of the lateral stabilizers [34]. The anconeus muscle mainly acts as an active posterolateral restraint rather than an active elbow extensor [28,30,45].

In cases involving PLRI and a fully ruptured and withdrawn CEO, the expected quality of the lateral structures’ soft tissues is poor. This limits the possibilities for any soft tissue re-tensing procedures [10]. Furthermore, due to retraction, arthroscopic repair is not a viable option. Therefore, reconstructing the LUCL using an autograft seems to be a reliable alternative [2]. When it comes to graft fixation, careful consideration must be given to the isometric point on the humerus, the ulnar insertion point, and the appropriate graft tension to reduce the risk of failure [2,10]. The isometric point can be found using an anatomical and radiological method [2], and careful intraoperative testing of the expected graft tension is essential [10]. Importantly, the natural LUCL does not follow a straight line in three dimensions. Consequently, implanting a straight-lined graft may cause it to stretch and potentially jeopardize the reconstruction [39,40]. Furthermore, simultaneous osseous constraint lesions must also be properly addressed to reduce the risk of ligament reconstruction failure [10]. Although LUCL reconstruction with a tendon autograft restores the elbow’s posterior lateral rotational stability, it does not always restore varus stability due to a potentially unchecked RCL dysfunction [39,40]. However, repairing the CEO after LUCL reconstruction may provide sufficient restoration of stability in varus [28].

The selection of the graft depends on the surgeon’s preference. Autografts are superior to allografts, apart from donor-site morbidity [46]. Thus, it is essential that autograft harvesting is a feasible choice in the surgeon’s armamentarium. Autograft options include the gracilis, the semitendinosus, the palmaris longus, the half-flexor carpi radialis, and the fascia lata, while all of them are strong enough to support the reconstruction [2,16,35,44]. It is the leading author’s (C.K.) preference to harvest triceps tendon autografts. Postoperative stability rates are reported over 90% [39]. According to recently published data, recurrent instability ranges from 0 to 33% for LCL repair and reconstruction surgery [10]. Nevertheless, to our knowledge, there are no data regarding postoperative stability rates for LUCL reconstruction with triceps autografts in complete rupture with retraction of the CEO. Incorporating this algorithm into upcoming research protocols would have the potential to address this deficit in the existing literature.

The advancement in arthroscopic techniques in the upper limb offers substantial benefits to both the surgeon and the patient. Open procedures that address the lateral aspect of the elbow often fail to preserve the CEO, hindering shorter rehabilitation programs and delaying the return to work or sports [47]. By using arthroscopy, an all-round examination of the elbow joint can be performed before the main procedure. This method helps identify any concealed pathology [47]. While some researchers discourage arthroscopic treatment for elbow instability due to potential nerve damage (ulnar or radial) [47], a recent systematic review revealed that arthroscopic treatment yields equivalent outcomes to combined arthroscopy and open procedures, with fewer complications and minimal soft tissue injury [14]. This discovery is crucial, especially since a cadaveric study showed that the adverse effects of open techniques on the elbow, specifically regarding iatrogenic PLRI, are irreversible [38]. Therefore, managing PLRI through an open approach only seems beneficial when dealing with a CEO rupture with retraction.

Limitations of this study are present. Firstly, the accuracy of clinical tests to diagnose PLRI is patient- and examiner-dependent. This may lead to bias as minor instability might be underdiagnosed. However, in our proposed protocol, PLRI was confirmed by an arthroscope in all cases. There is no measurement of triceps brachii muscles preoperatively and before autografts are received, but the literature mentions no clinical reduction in muscle strength. Surgeons following this algorithm should be experienced arthroscopists. Another issue is the variability in the patient population and their demands. The postoperative protocol plays a significant role, and patient compliance can greatly affect the results. The primary limitation of this study is the lack of quantitative results. While our initial findings appear promising, the follow-up only extended to a maximum of 30 months. We would prefer to have a longer postoperative period for all participants before publishing the results. This would allow us to identify possible complications such as donor-site morbidity, the development of elbow arthritis, or a recurrence of instability. We advocate that arthroscopic surgeons use our treatment protocol on a larger scale as a tool for further research. It is not technically demanding for an experienced upper limb orthopedic surgeon and leverages existing knowledge. Multicenter studies could potentially uncover additional issues and suggest further improvements.

## 7. Conclusions

PLRI is a clinical entity that appears often in general practice, often masked under the veil of lateral epicondylitis. This treatment algorithm is intended for arthroscopic surgeons seeking to diagnose and treat an unstable elbow with as few complications as possible using minimally invasive techniques specific to each patient’s condition. Based on the CEO’s integrity, different techniques, or combinations thereof, may be applied, resorting to an open approach only in the most extreme cases where the CEO is completely torn and retracted (open LUCL reconstruction with graft). No or moderate lesions of the CEO could be treated with arthroscopic LCL imbrication, while extensive partial lesions or complete lesions without retraction of CEO are arthroscopically dealt with using plication and LCL imbrication. This proposed algorithm facilitates not only preoperative but also intraoperative planning with simultaneous avoidance of open surgery and possible complications. In every case, a preoperative MRI is mandatory to examine the CEO’s integrity. Further research, including thorough follow-ups and optionally patient-reported outcome measure scores, is necessary.

## Data Availability

No new data were created or analyzed in this study. Data sharing is not applicable to this article.
